# QTc Interval and Its Associated Factors in Patient With Type 2 Diabetic Mellites

**DOI:** 10.1155/jdr/8664620

**Published:** 2025-12-01

**Authors:** Deresse Sinamaw Asmare, Tadegew Adane Abebe, Baye Ashenef, Temesgen Baylie, Yeshimareg Shita

**Affiliations:** Biomedical Science, Debre Markos University College of Health Science, Debre Markos, Amhara, Ethiopia

**Keywords:** associated factors, Ethiopia, QT interval, Type 2 diabetes

## Abstract

**Introduction:**

There is a notable link between diabetes mellitus (DM) and cardiovascular diseases (CVDs), particularly abnormal ventricular repolarization marked by a prolonged QT interval, which significantly contributes to morbidity and mortality in diabetic patients. However, no studies have been conducted in Ethiopia regarding prolonged QT intervals in Type 2 diabetic patients. Therefore, this study is aimed at examining the prevalence of prolonged QT intervals and the associated factors among Type 2 diabetic patients in selected referral hospitals in the Amhara Region, Ethiopia.

**Methods:**

A cross-sectional study was conducted from May 30, 2024, to September 30, 2024, involving 300 participants. Participants were selected using systematic sampling. Data were collected through questionnaires, anthropometric measurements, blood pressure readings, and lipid profile analyses. QTc interval was determined by using digital electrocardiographic machine. The study utilized bivariable and multivariable logistic regression models to identify factors associated with prolonged QT intervals.

**Results:**

The average QT interval was 420 ± 4.8 ms, with 45.6% of patients exhibiting prolonged QT intervals. Multivariable logistic regression analysis identified significant associations between prolonged QT intervals and factors such as age (AOR 1.11, 95% CI: 1.01–2.42), duration of diabetes (AOR 2.52, 95% CI: 1.12–5.31), body mass index (AOR 1.65, 95% CI: 1.21–3.44), fasting blood sugar (AOR 2.21, 95% CI: 1.12–6.21), sulfonylureas drug treatment (AOR 2.42, 95% CI: 1.21–5.94), HbA1c (AOR 2.78, 95% CI: 1.07–6.48), and total cholesterol (AOR 6.21, 95% CI: 4.25–11.25).

**Conclusion:**

This study highlights the significant prevalence of prolonged QT intervals among Type 2 diabetic patients. Several factors significantly contribute to the likelihood of prolonged QT intervals in Type 2 diabetic patients. These include older age, longer duration of diabetes, higher BMI, elevated fasting blood sugar levels, use of sulfonylureas, higher HbA1c levels, and elevated total cholesterol. This significant finding underscores the importance of regular cardiovascular monitoring and effective management strategies to mitigate the risks associated with prolonged QT intervals in this population.

## 1. Introduction

There is a significant connection between diabetes mellitus (DM) and cardiovascular diseases (CVDs), which is a major contributor to illness and death among diabetic patients [1–3]. This relationship is intricate and involves multiple factors such as autonomic dysfunction, changes in the structure of the heart's atria and ventricles, and various molecular changes [3]. Furthermore, conditions like abnormal blood sugar levels, abnormal lipid levels, and high insulin levels alter the metabolic profiles and cellular signaling within the cardiovascular system [4–6].

Cardiac autonomic neuropathy (CAN) is common among diabetic patients and heightens the risk of heart rhythm disorders and events like sudden death and heart attacks [7–10]. The severity of CAN in diabetic patients is linked to QTc prolongation [11–15]. The primary causes of QTc prolongation include chronic diabetes, ischemic heart disease, and autonomic system failure, with less common causes being water and electrolyte imbalances [16–18].

The QT interval represents the time required for the ventricles of the heart to undergo depolarization and repolarization [19–21]. Various physiological factors, including age, sex, heart rate, and autonomic system activity, influence the duration of the QT interval. Consequently, the QT interval is adjusted for the heart rate, resulting in the QTc interval [22–24].

Studies have shown that the prevalence of prolonged QTc intervals among Type 2 diabetic patients is 44.1% in Serbia [25], 25.8% in Italy [26], 30.1% in China [27], and 13% in Saudi Arabia [28]. A study conducted in Serbia found that prolonged QTc interval was significantly associated with female gender, high blood glucose, and sulfonylureas treatment [25]. In a similar study conducted in Italy, age and sex were significantly associated with QT prolongation [26]. In China, high glucose and height (Ht) were significantly associated with prolonged QT interval [27]. Despite the lack of studies on this topic in Ethiopia, this research is aimed at determining the prevalence of prolonged QT interval and its associated factors among patients with Type 2 diabetes.

## 2. Methods and Materials

### 2.1. Study Design, Study Area, and Setting

A cross-sectional study was conducted among Type 2 diabetic patients at selected referral hospitals in Amhara Region, Ethiopia, including Debre Tabor Referral Hospital, Gondar University Specialized Hospital, and Debre Markos Referral Hospital, from May 30, 2024, to September 30, 2024.

### 2.2. Sample Size Determination and Sampling Technique

The sample size was calculated using the single population proportion calculation, assuming a 95% confidence interval (CI) and a 5% margin of error (*d* = 0.05), based on an estimated prolonged QTc prevalence of 25% (*p* = 0.25) from a Ugandan study [29]). The overall sample size was 300, with a 5% nonresponse rate. The study comprised three of the Amhara Region's seven referral hospitals that were chosen at random. Samples were allocated proportionally based on the number of T2DM patients at each facility. Participants were then recruited from the selected hospitals using a systematic sampling technique. As the starting point, a lottery method was utilized to randomly select two numbers from a range of 1–6. The sixth participant was chosen for follow-up.

### 2.3. Data Collection Process

Data collection was carried out using a questionnaire adapted from the WHO STEP-wise approach to chronic disease risk factor surveillance [30]. After obtaining informed consent from the participants, sociodemographic data, behavioral factors, anthropometric measurements, blood glucose, and blood pressure (BP) readings were gathered from the study participants.

### 2.4. Anthropometric Measurement

The study participants' weight and Ht were measured to the nearest 100 g and 1 mm, respectively, with a digital scale and associated Ht gauge. Waist circumference (WC) and Ht were measured to the nearest 0.1 cm with a flexible, nonstretchable tape.

### 2.5. BP Measurement

Nurse professionals measured BP using a mercury sphygmomanometer. After participants rested for at least 5 min, BP was taken twice from the left arm at 5-min intervals. The average of these two readings was then calculated for analysis. Clinical nurses collected all data and measurements under the strict supervision of the investigators.

### 2.6. Lipid Profile Measurement

A laboratory technologist drew about 5 mL of blood from each participant after they had fasted overnight. The serum was then separated from the whole blood, and serum lipid profiles, including total cholesterol (TC) and triglycerides (TGs), were analyzed at the central laboratory. These analyses were performed using a clinical chemistry analyzer, the Siemens Dimension EXL 200 system, following standard protocols.

### 2.7. Fasting Blood Glucose Measurement

Fasting blood glucose was measured by taking a blood sample from all participants by using a glucometer. Impaired fasting blood glucose is defined when > 125 mg/dL.

### 2.8. Electrocardiographic Measurement

The study used standard resting 12 lead digital electrocardiographic machines with the model of CONTEC ECG 1200G (made in China) with a paper speed of 25 mm per second calibrated on 1 mv = 10 mm, 0.2 s = 5 mm, where each large box and the small box represents 0.2 and 0.04 s, respectively. Measurement will be performed at a supine position with a 45° inclination after the patient had been asked to expose the chest region and removing electromagnetic objects on his/her body. And, orientation will be given for the patients to stop speaking, moving, and reduce breathing during ECG recording. Then, 10 electrodes (four limb electrodes at right arm and left arm and legs and six chest electrodes, V1–V6) will be placed on the participant's arms, legs, and chest after the transparent gel applied, yields a total of 12 leads. Finally, to identify ECG tracings as having an abnormality, the Minnesota coding system will be used [31].

### 2.9. Operational Definition

**Prolonged QT** **interval** is when complete depolarization and repolarization period takes greater than 440 ms. [32]

**Body mass index** (BMI) was classified into four categories: underweight (BMI < 18.5 kg/m^2^), normal weight (18.5–24.9 kg/m^2^), overweight (25–29.9 kg/m^2^), and obese (BMI ≥ 30 kg/m^2^) [33].

### 2.10. Statistical Data Analysis

The data collected were reviewed and entered into Epi-Data Version 4.6 and then exported to STATA Version 4.6 for analysis. Descriptive analysis was conducted, with results presented in tables. Categorical variables were analyzed using frequency and percentage, while continuous variables were expressed as mean ± standard deviation (SD) and median (interquartile range (IQR)). The chi-square test was used to compare categorical variables. The Shapiro–Wilk test assessed the normality of data distribution. To explore relationships between outcome variables and potential associated factors, bivariable and multivariable binary logistic regression models were used. Variables with *p* values < 0.25 in bivariable logistic regression were included in the multivariable logistic regression model for final analysis. Crude odds ratio (COR) and adjusted odds ratio (AOR) with 95% CI were reported, with a *p* value < 0.05 considered statistically significant. Multicollinearity among selected independent variables was checked, and the variance inflation factor was acceptable (less than 5). The Hosmer and Lemeshow test determined the goodness-of-fit of the model.

## 3. Results

### 3.1. Sociodemographic Characteristics

In this study, 300 patients with Type 2 diabetes participated, with an average age of 49.2 ± 9.3 years. Of these, 60% (180 patients) were female and 65.3% (196 patients) resided in urban areas. Additionally, 42.7% (128 patients) were unemployed. The study found that 42.7% of the patients had inadequate physical activity and 66% (198 patients) were married (Table 1).

### 3.2. Anthropometric and Biochemical Characteristics of Study Participants

Among the study participants, 59.7% (179 out of 300) had a BMI over 25 kg/m^2^, showing a significant difference in QT interval duration between those with intervals greater than 440 ms and those with less than 440 ms. Additionally, 67.7% were hyperglycemic, with fasting blood sugar levels above 130 mg/dL, which also significantly differed between the two QT interval groups. Sulfonylurea exposure was noted in 52% of participants, showing a significant difference in prolonged versus normal QT intervals. Furthermore, 31.7% of participants had HbA1c levels exceeding 6.5%, which was associated with significant differences in both QT interval duration and TC levels between those with prolonged QT intervals and those with normal QT intervals (Table 2).

### 3.3. Prolonged QT Interval Distribution

The average QT interval among Type 2 diabetic patients was 420 ± 4.8 ms. Additionally, 45.6% of these patients had a prolonged QT interval.

### 3.4. Factors Associated With Prolonged QT Interval in Type 2 Diabetic Patients

The multivariable logistic regression analysis revealed that age, duration of diabetes, body mass index, fasting blood sugar levels, type of diabetes medication, HbA1c levels, and TC were all significantly associated with prolonged QT intervals. Each one-unit increase in age raised the likelihood of a prolonged QT interval by 1.1 times (AOR 1.11, 95% CI: 1.01–2.42). Patients with diabetes for more than 10 years were 2.52 times more likely to have a prolonged QT interval compared to those with less than 2 years of diabetes (AOR 2.52, 95% CI: 1.12–5.31). A BMI over 25 kg/m^2^ increased the likelihood of a prolonged QT interval by 1.65 times (AOR 1.65, 95% CI: 1.21–3.44). Patients with fasting blood sugar levels above 130 mg/dL were 2.21 times more likely to develop a prolonged QT interval compared to those with levels below 130 mg/dL (AOR 2.21, 95% CI: 1.12–6.21). Those taking sulfonylureas were 2.42 times more likely to have a prolonged QT interval compared to those on metformin (AOR 2.42, 95% CI: 1.21–5.94). Patients with HbA1c levels above 6.5% were 2.78 times more likely to have a prolonged QT interval compared to those with levels below 6.5% (AOR 2.78, 95% CI: 1.07–6.48). Patients with TC levels above 200 mg/dL were 6.21 times more likely to have a prolonged QT interval compared to those with levels below 200 mg/dL (AOR 6.21, 95% CI: 4.25–11.25) (Table 3).

## 4. Discussion

The purpose of this study was to determine the prevalence of prolonged QT intervals and the factors that contribute to them in Type 2 diabetic individuals. Notably, this is the first study of its kind in Ethiopia. The prevalence of prolonged QT interval was 45.6% which is higher than the study conducted in Serbia 44.1% [25], Italy 25.8% [26], China 30.1% [27], Saudi Arabia 13% [28], and Uganda (29.8%) [29]. However, the prevalence of prolonged QT interval was lower than the study conducted in Nigeria (52.14%) [34]. The variation might be due to the difference in lifestyle such as level of physical activity, type of diabetic medication used, glycemic control, and dyslipidemia management practice [35–37].

Prolonged QT interval in Type 2 diabetic patients can indeed be influenced by several factors. These include age, duration of diabetes, body mass index, fasting blood sugar levels, type of diabetes medication, HbA1c levels, and TC.

The likelihood of developing prolonged QT interval in older patients with Type 2 diabetes mellitus was by 1.1 times higher as compared to younger T2DM patients. This study finding is consistent with the study conducted in Saudi Arabia [28], Italy [26], and China [27]. In older patients, the increased prevalence of atrial and ventricular arrhythmias can be attributed to several factors. Aging disrupts the normal sequence of ion channel activation and inactivation, which is crucial for generating the cardiac action potential. Additionally, aging brings about structural changes, such as myocardial fibrosis, which further contributes to the prolongation of the QT interval. These biochemical, electrophysiological, and structural changes collectively increase the risk of arrhythmias and QT interval prolongation in older adults [38–41].

The risk of a prolonged QT interval in Type 2 diabetic patients is significantly higher for those with a diabetes duration of more than 10 years, being 2.52 times greater compared to those who have had diabetes for less than 2 years. Therefore, this study was in line with the study conducted in Saudi Arabia [28] and China [27]. A prolonged duration of diabetes can lead to diabetic cardiomyopathy, severe coronary atherosclerosis, prolonged hypertension, chronic hyperglycemia, microvascular disease, glycosylation of myocardial proteins, and autonomic neuropathy. These conditions contribute to CVD, which results in abnormalities in ventricular depolarization and repolarization [42–45].

In this study, the prolonged QT interval in Type 2 diabetic patients with a BMI greater than 25 kg/m^2^ was found to be 1.65 times higher compared to those with a BMI less than 25 kg/m^2^. This study was consistent with study conducted in China [27] and inconsistent with the study conducted in Serbia [25] and Saudi Arabia [28]. This could be attributed to the fact that obesity is linked to insulin resistance, dyslipidemia, hypertension, and other conditions that contribute to CVD, which may specifically prolong the time of repolarization [46–49].

This study indicates that the prolonged QT interval in Type 2 DM patients with hyperglycemia (fasting blood glucose > 130 mg/dL) was 2.21 times higher compared to those with fasting blood glucose levels below 130 mg/dL. These findings are consistent with a similar study conducted in Serbia [25] and China [27]. The activation of nonoxidative glucose pathways (NOGPs) due to hyperglycemia, including the polyol pathway, hexosamine biosynthetic pathway, advanced glycation end products (AGEs), and protein kinase C, plays a significant role in the development of CVD. This, in turn, can lead to a prolonged QT interval [50–52]. Type 2 diabetic patients with HbA1c levels above 6.5% are more likely to develop prolonged QT intervals compared to those with HbA1c levels below 6.5%. This finding is consistent with a study conducted in China [27] but contradicts results from a study in Saudi Arabia [28]. High HbA1c levels can indeed elevate oxidative stress and inflammation, which negatively affect the heart's electrical conduction system. This can lead to disruptions in the heart's normal rhythm, potentially causing prolonged QT intervals and increasing the risk of arrhythmias [53, 54].

Patients with Type 2 diabetes who used sulfonylureas were more prone to experiencing a prolonged QT interval than those who used metformin or insulin. This finding aligns with a study from Serbia [25]. However, this contradicts a study from Saudi Arabia that identified a significant link between insulin treatment and a prolonged QT interval. This discrepancy might be because sulfonylureas has various effects. Firstly, they cause weight gain, a known risk factor for CVD. Secondly, these drugs can lead to fluid retention, raising the risk of heart failure. Thirdly, severe hypoglycemia, a potential side effect of sulfonylureas, can trigger cardiac arrhythmias and other cardiovascular events. Lastly, sulfonylureas may disrupt the heart's natural protective mechanisms, potentially leading to increased cell death and arrhythmias [55–57].

Type 2 diabetic patients with TC levels above 200 mg/dL are 6.21 times more likely to experience prolonged QT intervals compared to those with levels below 200 mg/dL. This finding aligns with studies conducted in Serbia [25] and China [27]. TC can affect the QT interval in DM through various mechanisms. Diabetes often causes an imbalance in lipid levels, known as diabetic dyslipidemia, which includes high TC and TGs and low HDL cholesterol. This imbalance can lead to cardiovascular issues, including QT prolongation [28, 58]. High cholesterol levels can also increase inflammation and oxidative stress, impacting the heart's electrical activity and potentially prolonging the QT interval [59, 60]. Additionally, elevated cholesterol can contribute to atherosclerosis, impairing coronary blood flow and affecting the heart's electrical conduction system [61, 62]. Cholesterol can directly influence ion channel function in the heart, which is essential for maintaining a normal QT interval. Changes in ion channel function can disrupt the heart's electrical activity, leading to QT prolongation [60, 63].

## 5. Conclusion

This study highlights the significant prevalence of prolonged QT intervals among Type 2 diabetic patients. Several factors significantly contribute to the likelihood of prolonged QT intervals in Type 2 diabetic patients. These include older age, longer duration of diabetes, higher BMI, elevated fasting blood sugar levels, use of sulfonylureas, higher HbA1c levels, and elevated TC. This significant finding underscores the importance of regular cardiovascular monitoring and effective management strategies to mitigate the risks associated with prolonged QT intervals in this population.

## Figures and Tables

**Table 1:** Sociodemographic characteristics of Type 2 diabetic patients in selected referral hospitals of Amhara regions, Northwest Ethiopia, 2024 (*n* = 300).

**Variables**	**Categories**	**Total (%)** **(** **N** = 300**)**	**QT duration**		**X** ^2^ **p value**
			**< 440 ms (%)**	**> 440 ms (%)**	
Sex	Male	120 (40)	62 (51.7)	58 (48.3)	*p* = 0.215
	Female	180 (60)	78 (43.3)	102 (56.7)	

Age (years)	< 45	75 (25)	9 (12)	66 (88)	*p* < 0.05
	46–54	52 (17.3)	12 (23.1)	40 (76.9)	
	55–64	72 (24)	21 (29.2)	51 (70.8)	
	> 65	111 (37)	32 (28.8)	79 (71.2)	

Residence	Urban	196 (65.3)	143 (73)	53 (27)	*p* = 0.254
	Rural	102 (34)	81 (79.4)	21 (20.6)	

Current marital status	Married	198 (66)	152 (76.8)	46 (23.2)	*p* = 0.574
	Unmarried	102 (33)	87 (85.3)	15 (14.7)	

Educational status	Illiterate	102 (34)	71 (69.6)	31 (30.4)	*p* = 0.245
	Literate	198 (66)	112 (56.6)	86 (43.4)	

Occupation	Employed	172 (57.3)	60 (34.9)	112 (65.1)	*p* = 0.004
	Unemployed	128 (42.7)	50 (39)	78 (61)	

Regular exercise	Yes	172 (57.3)	110 (64)	62 (36)	*p* = 0.687
	No	128 (42.7)	58 (45.3)	70 (54.7)	

*Note:* Continuous variables are expressed in mean (SD), and categorical variables are expressed as number and percentage.

**Table 2:** Anthropometric and biochemical characteristics of Type 2 diabetic patients in selected referral hospitals of Amhara regions, Northwest Ethiopia, 2024 (*n* = 300).

**Variables**	**Total %** **(** **N** = 300**)**	**QT duration**		**X** ^2^ **p value**
		**< 440 ms**	**> 440 ms**	
Body mass index (kg/m^2^)				*p* < 0.05
< 25	121 (40.3)	37 (30.1)	84 (69.9)	
25 and above	179 (59.7)	48 (26.8)	131 (73.2)	
FBS				*p* < 0.05
<130 mg/dL	97 (32.3)	55 (56.7)	42 (43.3)	
≥130 mg/dL	203 (67.7)	64 (31.5)	139 (68.5)	
Type of DM drug				*p* < 0.05
Metformin	99 (33)	54 (54.5)	45 (45.5)	
Insulin	45 (15)	21 (46.7)	24 (53.3)	
Sulfonylureas	156 (52)	54 (34.6)	102 (65.4)	
HbA1c				*p* < 0.05
< 6.5%	205 (68.3)	106 (51.7)	99 (48.3)	
> 6.5%	95 (31.7)	25 (26.3)	70 (73.7)	
Total cholesterol				*p* < 0.05
< 200 mg/dL	166 (55.3)	112 (67.5)	54 (32.5)	
> 200 mg/dL	134 (44.7)	22 (16.4)	112 (83.6)	
Triglyceride				*p* = 0.65
< 150 mg/dL	200 (66.7)	101 (50.5)	99 (49.5)	
> 150 mg/dL	100 (33.3)	54 (54.0)	46 (46.0)	

*Note:* Continuous variables are expressed in mean (SD), and categorical variables are expressed as number and percentage.

Abbreviation: *N*, number.

**Table 3:** Factors associated with prolonged QT interval in Type 2 diabetic patients in selected referral hospitals of Amhara regions, Northwest Ethiopia, 2024 (*n* = 300).

**Variable**	**QTc duration**		**COR (95% CI)**	**AOR (95% CI)**
	**< 440 ms**	**> 440 ms**		
Age in years			1.25 (1.09, 2.56)	1.11 (1.01, 2.42)⁣^∗^
Age in group				
< 45	9 (12)	66 (88)	1	1
46–54	12 (23.1)	40 (76.9)	1.55 (0.44, 6.65)	1.21 (0.54, 6.34)
55–64	21 (29.2)	51 (70.8)	2.74 (1.22, 8.45)	2.12 (0.71, 7.50)
> 65	32 (28.8)	79 (71.2)	3.1 (1.28, 10.21)	2.5 (1.11, 10.24)⁣^∗^
Sex				
Female	78 (43.3)	102 (56.7)	0.45 (0.75, 2.25)	0.23 (0.11, 9.16)
Male	62 (51.7)	58 (48.3)	1	1
Occupation				
Employed	60 (34.9)	112 (65.1)	1	1
Unemployed	50 (39)	78 (61)	1.57 (0.51–2.12)	0.46 (0.11, 1.06)
Duration of DM in (years)				
< 2	15 (38.5)	24 (61.5)	1	1
2–5	65 (55.6)	52 (44.4)	1.1 (0.56, 2.54)	1.04 (0.40, 2.37)
6–10	42 (65.6)	22 (34.4)	1.14 (0.72, 3.62)	0.42 (0.11, 1.28)
> 10	13 (16.3)	67 (83.7)	3.45 (2.64, 6.11)	2.52 (1.12, 5.31)⁣^∗^
Body mass index (kg/m^2^)				
< 25	37 (30.1)	84 (69.9)	1	1
25 and above	48 (26.8)	131 (73.2)	2.12 (1.31, 4.24)	1.65 (1.21, 3.44)⁣^∗^
FBS				
< 130 mg/dL	55 (56.7)	42 (43.3)	1	1
≥ 130 mg/dL	64 (31.5)	139 (68.5)	3.72 (1.45, 7.45)	2.21 (1.12, 6.21)⁣^∗^
Type of DM drug				
Metformin	54 (54.5)	45 (45.5)	1	1
Insulin	21 (46.7)	24 (53.3)	1.11 (0.98, 2.54)	1.05 (0.75, 2.14)
Sulfonylureas	54 (34.6)	102 (65.4)	2.65 (1.45, 6.58)	2.42 (1.21, 5.94)⁣^∗^
HbA1c				
< 6.5%	106 (51.7)	99 (48.3)	1	1
> 6.5%	25 (26.3)	70 (73.7)	3.25 (1.12, 8.47)	2.78 (1.07, 6.48)⁣^∗^
Total cholesterol				
< 200 mg/dL	112 (67.5)	54 (32.5)	1	1
> 200 mg/dL	22 (16.4)	112 (83.6)	10.52 (5.42, 14.50)	6.21 (4.25, 11.25)⁣^∗^
Triglyceride				
< 150 mg/dL	101 (50.5)	99 (49.5)	1	1
> 150 mg/dL	54 (54.0)	46 (46.0)	1.42 (0.78, 3.54)	1.11 (0.56, 2.48)

*Note:* 1 = constant. Hosmer–Lemeshow goodness-of-fit *p* = 0.31.

Abbreviation: FBS, fasting blood sugar.

⁣^∗^*p* < 0.05.

## Data Availability

The datasets generated and/or analyzed during the current study are available from the corresponding author on reasonable request.

## Nomenclature

BMI: body mass index
CVD: cardiovascular disease
T2DM: Type 2 diabetes mellitus
ECG: electrocardiogram
FBS: fasting blood sugar
